# The effect of chronic lymphocytic thyroiditis on patients with thyroid cancer

**DOI:** 10.1186/1477-7819-12-277

**Published:** 2014-09-01

**Authors:** Yi Zhang, Xiao-peng Ma, Fu-sheng Deng, Zheng-rong Liu, Hou-qing Wei, Xi-hong Wang, Hao Chen

**Affiliations:** Department of General Surgery, The Affiliated Provincial Hospital of Anhui Medical University, No.1, Tian’ehu Road, 230001 Hefei, China

**Keywords:** Chronic lymphocytic thyroiditis, Thyroid malignancy, Prognosis

## Abstract

**Background:**

The purpose of this study was to investigate the association between chronic lymphocytic thyroiditis (CLT) and malignant tumors of the thyroid.

**Methods:**

A retrospective review of 647 patients who underwent thyroid surgery at the Department of Breast and Thyroid Surgery in Anhui Provincial Hospital, China in 2012 was performed. The clinicopathological characteristics of patients with thyroid malignancies and CLT were collected. CLT was diagnosed by histopathological method.

**Results:**

Among 647 patients, 144 patients had thyroid malignancies and 108 patients had been diagnosed with CLT. Moreover, in total, 44 patients had thyroid malignancies coexistent with CLT: forty-one (93.2%) patients had been diagnosed with the papillary thyroid cancer (PTC); two (4.5%) patients suffered from medullary carcinoma; and one (2.3%) patient suffered from lymphoma. The morbidity of thyroid malignancies in patients with CLT was significantly higher than that in patients without CLT (40.7% versus 18.6%; *P* <0.001). A female preponderance was observed in the patients with CLT compared with those without CLT (*P* <0.001). There was no statistically significant difference in the tumor size (*P =* 0.073), multifocality (*P* = 0.0871), neck lymph node metastasis (*P* = 0.350), age (*P* = 0.316), microcarcinoma (*P* = 0.983) and tumor-node-metastasis (TNM) stage (*P* = 0.949) between the patients of thyroid malignancies with CLT and without CLT.

**Conclusions:**

Female predominance was observed in patients with CLT. CLT may have no effect on the progression of thyroid malignant tumor. Nevertheless, the influences of CLT on the prognosis of the thyroid carcinoma still need to be investigated with a larger sample size.

## Background

Thyroid cancer is the most common endocrine malignancy and occurs in 8.7 per 100 000 individuals in the United States, accounting for approximately 1% of all cancers [1–3]. It has been reported that clinicopathologic factors, such as sex, age, tumor size, multifocality, extrathyroidal extension and lymph node metastasis, are related to the prognosis of thyroid cancers [4–6]. Different morphological types of thyroid cancer mainly include papillary thyroid carcinoma (PTC), follicular carcinoma, medullary carcinoma, anaplastic carcinoma and thyroid lymphoma. PTC is the most prevalent manifestation of thyroid cancer. It represents 70 to 80% of all diagnosed thyroid cancers [7]. Thyroid lymphoma is a rare thyroid malignant tumor, accounting for 1 to 5% of all thyroid malignancies and less than 2% of extranodal lymphomas [8].

Chronic lymphocytic thyroiditis (CLT, or Hashimoto's thyroiditis (HT)) has been recognized as a common autoimmune thyroid disorder in which the thyroid gland is attacked by various antibody and cell-mediated immune processes [9]. The clinical and pathologic associations between CLT and thyroid epithelial neoplasms were first described by Dailey *et al.* in 1955 [10]. Studies have found that CLT can coexist with other autoimmune thyroid diseases and thyroid malignancies [11, 12]. A well-established association that patients with CLT have an increased risk of thyroid lymphoma and PTC has been proposed [13–16]. In addition, Okayasu *et al.* [17] revealed an association between CLT and PTC in the Japanese, and in white and African American populations, and also indicated that the possibility of CLT as a predisposing factor for PTC. However, the association between PTC and CLT with regard to pathogenesis and prognostic outcomes of their co-occurrence remains unclear among Chinese population; furthermore, the association between the other types of thyroid cancers and CLT still needs to be studied.

In this present study, we aimed to evaluate the presence of coexistent CLT in a population of patients who underwent thyroid surgery at the Department of Breast and Thyroid Surgery in Anhui Provincial Hospital, China. Then the association of CLT with clinicopathological parameters including age, sex, tumor size, multifocality, neck lymph node metastasis, microcarcinoma and TNM stage of patients with thyroid malignancies was determined.

## Methods

### Patients

In 2012, 647 patients who had undergone thyroid surgery at the Department of Breast and Thyroid Surgery in Anhui Provincial Hospital, China were enrolled in this study. All participants gave written informed consents at the time of surgery to allow future reviewing of medical records, and this retrospective study was approved by the hospital medical ethics committee.

### Diagnosis of chronic lymphocytic thyroiditis

The histopathological manifestations of CLT include diffuse lymphoplasmacytic and plasma cell infiltration, lymphoid follicle formation with germinal centers, varying degrees of fibrosis, parenchymal atrophy in the area of normal thyroid tissue, and the presence of large follicular cells with abundant oxyphilic cells. Only the presence of peri-tumoral lymphocytic infiltration was not considered as CLT [18–20].

### Data collection

The general characteristics of the patients with thyroid malignancies were recorded, including age, sex and cancer types. The clinicopathological parameters, such as tumor size, multifocality, neck lymph node metastasis, microcarcinoma and TNM stage, of patients with thyroid malignancies and with and without CLT were collected to analyze the effect of coexistent CLT in the patients with thyroid cancers.

Meanwhile, the serological examination results, preoperative ultrasonographic examination results and surgical methods of patients of thyroid malignancies with coexistent CLT were collected.

### Statistical analysis

Statistical analysis was performed by SPSS 11.5 software (SPSS, Chicago, IL, USA). The measurement data were expressed as mean ± standard deviation and analyzed by *t*-test. The chi-square (*χ*^2^) test was used to analyze the enumeration data. There is significantly statistical difference when *P* <0.05.

## Results

### General characteristics of patients with thyroid malignancies

The general characteristics of patients with thyroid malignancies are shown in Table 1. Among the 647 patients who underwent thyroid surgery, there were 144 patients (female/male: 106/38; 46.9 ± 13.9 years) with thyroid malignancies, 108 patients (female/male: 101/7) with CLT and 395 with other thyroid diseases. Moreover, 44 patients (female/male: 41/3; 43.3 ± 13.2 years) with thyroid malignancies had co-existing CLT. Among them, 41 patients had been diagnosed with papillary carcinoma, 2 patients suffered from medullary carcinoma and 1 patient developed lymphoma.

**Table 1:** **The general characteristics of patients with thyroid malignancies and the morbidity of malignancy of patients with or without chronic lymphocytic thyroiditis (CLT)**

		CLT(+) (n = 108)	CLT(-) (n = 539)
Malignancy (-) (n = 503)		64 (59.3%)	439 (81.4%)
Malignancy (+) (n = 144)		44 (40.7%)	100 (18.6%)
	Age (years)	43.3 ± 13.2	48.3 ± 14.1
	Sex (female/male)	41/3	65/35
	Papillary carcinoma	41 (93.2%)	93 (93.0%)
	Follicular carcinoma	0	2 (2.0%)
	Medullary carcinoma	2 (4.5%)	2 (2.0%)
	Anaplastic carcinoma	0	3 (3.0%)
	Lymphoma	1 (2.3%)	0

CLT, chronic lymphocytic thyroiditis; CLT (+), malignancy of patients with CLT; CLT (-), malignancy of patients without CLT.

### Comparison of morbidity of malignancy between patients with and without chronic lymphocytic thyroiditis

Comparison of morbidity of malignancy between patients with and without CLT were shown in Table 1. Thyroid malignancies were detected in 44 (40.7%) of the 108 patients with CLT and 100 (18.6%) of the 539 patients without CLT. The morbidity of thyroid malignancies between patients with CLT was significantly higher than patients without CLT (*P* <0.001).

### Clinicopathological parameters of patients with and without chronic lymphocytic thyroiditis

The differences in clinicopathological parameters between patients with and without CLT were analyzed in Table 2. The 144 patients with thyroid malignancies were mainly at stage I (83/144, 57.6%) and stage II (34/144, 23.6%). There was no statistically significant difference in the tumor size (*P* = 0.073), multifocality (*P* = 0.0871), neck lymph node metastasis (*P* = 0.350), age (*P* = 0.316), microcarcinoma (*P* = 0.983) and TNM stage (*P* = 0.949) between the patients of thyroid malignancies with CLT and without CLT. Female predominance was observed in patients with CLT compared to those without CLT (*P* < 0.001).

**Table 2:** **The differences in clinicopathological parameters between patients with and without chronic lymphocytic thyroiditis (CLT)**

Parameters	Malignancy (+) CLT (-)	Malignancy (+) CLT (+)	Total	P
	(n = 100)	(n = 44)	(n = 144)	
Tumor size (cm)				
d ≤2	59 (59.0%)	34 (77.3%)	93 (64.6%)	0.073
2 < d ≤4	28 (28.0%)	8 (18.2%)	36 (25.0%)	
d >4	13 (13.0%)	2 (4.5%)	15 (10.4%)	
Multifocality				
No	79 (79.0%)	36 (81.8%)	115 (79.9%)	0.0871
Yes	21 (21.0%)	8 (22.2%)	29 (20.1%)	
Neck lymph node metastasis				
No	65 (65.0%)	25 (56.8%)	90 (62.5%)	0.350
Yes	35 (35.0%)	19 (43.2%)	54 (37.5%)	
Gender				
Female	65 (65.0%)	41 (93.2%)	106 (73.6%)	<0.001
Male	35 (35.0%)	3 (6.8%)	38 (26.4%)	
Age				
<45 years	41 (41.0%)	22 (50.0%)	63 (43.7%)	0.316
≥45 years	59 (59.0%)	22 (50.0%)	81 (56.3%)	
Microcarcinoma				
No	68 (68.0%)	30 (68.2%)	98 (68.1%)	0.983
Yes	32 (32.0%)	14 (31.8%)	46 (31.9%)	
TNM stage				
I	57 (68.7%)	26 (31.3%)	83 (57.6%)	0.949
II	23 (67.6%)	11 (32.4%)	34 (23.6%)	
III	11 (73.3%)	4 (26.7%)	15 (10.4%)	
IVA	9 (75.0%)	3 (25.0%)	12 (8.3%)	

CLT, chronic lymphocytic thyroiditis; CLT (+), malignancy of patients with CLT; CLT (-), malignancy of patients without CLT; TNM stage, tumor-node-metastasis stage.

### Diagnosis results and surgical methods for patients of thyroid malignancies with coexistent chronic lymphocytic thyroiditis

There were 44 patients of thyroid malignancies with coexistent CLT, among which 37 patients had received serological examination. The level of serum thyroperoxidase antibody was elevated in 23 (62.2%) patients, along with the level of thyroglobulin antibodies in 32 (86.5%) patients. Preoperative ultrasonographic examination was applied to the 44 patients who had thyroid malignancies with coexistent CLT: nodular hyperplasia was detected in all patients and accounted for up to 100%, a solitary nodule was found in 15 (34.1%) patients, multiple nodules were present in 19 (65.9%) patients, spot calcification was found in 25 (56.8%) patients, irregular and unclear border appeared in 18 (40.9%) patients, and cervical lymph node enlargement were identified in 23 (52.3%) patients. Seven different surgical methods were assigned to these 44 patients who had thyroid malignancies and co-existing CLT. In total, 13 (29.5%) patients received a unilateral lobectomy plus central region lymph node dissection, and 10 (22.7%) patients received a unilateral lobectomy plus contralateral subtotal lobectomy with central region lymph node dissection. Moreover, a bilateral thyroidectomy plus central region lymph node dissection was assigned to six (13.6%) patients. In addition, 12 (27.3%) patients were equally allotted to 3 groups using bilateral thyroidectomy plus central region lymph node dissection, bilateral thyroidectomy plus unilateral neck dissection and bilateral thyroidectomy plus bilateral neck dissection. The other three (6.8%) patients received unilateral lobectomy plus unilateral neck dissection. All patients received endocrine therapy by means of oral drugs such as levothyroxine sodium, as well as the clinical follow-up after operation. The follow-up period was from 10 months to 22 months (average 15 months). There was no death or case recurrence.

## Discussion

The association between CLT and thyroid lymphoma and PTC has been well-established in Japan and America; however, the correlation between CLT and PTC in our country, as well as the influence of CLT on other thyroid malignancies, still needs to be studied. In our study, the association of CLT with clinicopathological parameters of patients with thyroid malignancies was evaluated. The results showed that the morbidity of thyroid malignancies in patients with CLT was significantly higher than that in patients without CLT. A female preponderance was observed in the patients with CLT compared with those without CLT. There was no statistically significant difference in the tumor size, multifocality, neck lymph node metastasis, age, microcarcinoma or TNM stage between the patients of thyroid malignancies with CLT and without CLT.

Among the 44 patients who had thyroid malignancies coexistent with CLT, 41 had been diagnosed with papillary carcinoma, 2 suffered from medullary carcinoma and 1 suffered from lymphoma. The results revealed that coexistence of CLT mainly occurred in the patients with PTC [21, 22], a result in accordance with other studies. In addition, we found that the rate of thyroid malignancies in patients with CLT (40.7%) is significantly higher than that in patients without CLT (18.6%). The presence of CLT has been reported to be in 0.5 to 38% of patients with PTC [17, 18, 23]. The presence of thyroid malignancies in patients with CLT in our study is slightly higher than that reported in the literature. The reasons may be multifactorial. First, the indications for surgery in our department are stricter, and the surgery was performed on patients with thyroid nodules that were highly suspected for thyroid cancer. Second, racial difference, genetic factors, and dietary factors might contribute to the result. Third, it should be note that the sample size of our study was relatively small compared with other studies, consisting of only 647 people. Some studies have reported that the prevalence of CLT is significantly increased in patients with PTC [24, 25]. Therefore, patients with thyroid nodules and who are suspected of having CLT need to be carefully monitored since the possibility of malignancy is raised. Once thyroid cancer is diagnosed, the most cost-effective approach should be chosen. Several treatment methods for patients with thyroid cancer, such as surgical resection, radioactive iodine ablation, and TSH suppression therapy, are all in favor [26]. However, controversy still exists in many areas. Accordingly, thyroidectomy is recommended for differentiated cancer (for example, papillary and follicular cancer) [7]. In contrast, for undifferentiated thyroid cancer (for example, anaplastic cancer) that is associated with aggression and invasion, chemotherapy and radiation treatment might provide more benefit to these patients compared to surgery [27]. In our study, among the 44 patients who had thyroid malignancies and co-existing CLT, papillary carcinoma was the main type of thyroid malignancy. Therefore, seven different surgical methods were assigned to these 44 patients. In addition, endocrine therapy, such as oral drugs, was provided to all patients after operation. Adjuvant therapy was also assigned to patients with extracapsular extensions or more lymph node metastasis. In our subsequent follow-up after operation, we found that there was no case of death or recurrence, possibly due to a good prognosis for thyroid malignancies and the short term of the follow-up.

In our study, a female predominance was observed in patients with CLT compared to those without CLT. Many previous studies have found that patients with CLT are predominately female in comparison with patients without CLT [28, 29]. This result is consistent with previous studies. However, no statistically significant difference was found in the tumor size, multifocality, neck lymph node metastasis, age, microcarcinoma and TNM stage between patients with thyroid malignancies and CLT and those patients with thyroid malignancies and no CLT. Generally speaking, immune infiltration in human tumors as a prognostic factor could not be ignored [30, 31]. CLT is characterized by progressive loss of thyroid epithelial cells and replacement of inflammatory cell infiltrate that produces chemokines, growth factors and cytokines [32–34]. Some studies have reported that the lymphocytic infiltration in PTC is associated with a lower recurrence rate, a better prognosis, and less aggressive disease [19, 35, 36]. However, Kebebew *et al*. have reported that thyroid lymphocytic infiltration is not an independent prognostic factor and could not reduce the recurrence rate or frequency of distant metastasis [23]. Among the 37 patients who received serological examination, we found that the level of serum thyroperoxidase antibody was high in 23 (62.2%) patients, as well as the level of thyroglobulin antibodies in 32 (86.5%) patients. Because thyroglobulin and thyroperoxidase are the two primary antigens in autoimmune thyroiditis, patients who suffered from CLT would make corresponding autoantibodies against thyroid-specific antigens [32, 37, 38]. It has been reported that serum thyroglobulin antibody was an independent predictor for thyroid malignancy in thyroid nodules [34, 39].

## Conclusions

In conclusion, we analyzed the association of CLT with clinicopathological parameters of patients with thyroid malignancies. The results of our study indicate that the CLT may have no effect on the progression of thyroid malignant tumor. Nevertheless, the influences of CLT on the prognosis of the thyroid carcinoma still need to be investigated based on a larger sample size.

## Abbreviations

CLT: chronic lymphocytic thyroiditis
HT: Hashimoto's thyroiditis
PTC: papillary thyroid carcinoma
TNM: tumor–node–metastasis.
